# Fungi Associated With Woody Tissues of European Beech and Their Impact on Tree Health

**DOI:** 10.3389/fmicb.2021.702467

**Published:** 2021-08-26

**Authors:** Gitta Jutta Langer, Johanna Bußkamp

**Affiliations:** Section Mycology and Complex Diseases, Department of Forest Protection, Northwest German Forest Research Institute (NW−FVA), Göttingen, Germany

**Keywords:** *Fagus sylvatica*, latent pathogens, virulence, pathogenicity, inoculation tests

## Abstract

Filamentous fungi associated with woody tissues of European Beech (*Fagus sylvatica*) and isolated from diseased trees and healthy trees were examined in relation to their impact on tree health. To this end, classical culture-based isolation methods, in planta inoculations and fungal identification using ITS-barcode and morphological characters were used. Stem endophytes of healthy beech saplings collected in German forests were isolated to determine endophyte communities in woody stem tissues. Pathogenicity tests were performed on living potted beech saplings using twelve selected fungal pathogens and wood inhabiting fungi (Hypocreales, Botryosphaeriales, and Xylariales) originating mainly from European beech with symptoms of the complex disease Vitality loss, or from bark necroses, or known to be common endophytes of beech. The impact of these ascomycetous fungi with respect to tree health was discussed. The potential influences of endophytic fungi of beech and of test conditions are discussed in relation to the success of inoculation. All tested fungal strains except for *Neonectria ditissima* were able to establish themselves post inoculation in the beech stems and caused necroses when there was sufficient water, but at different severities. Under the experimental conditions, *Botryosphaeria corticola* was shown to be the most virulent tested latent pathogen against *F. sylvatica*. In the context of climate change and global warming, the tested Botryosphaeriaceae are able to play a primary role in the disease progress of Vitality loss of Beech. The key role of *Neonectria coccinea* in causing bark necroses and the loss of vitality in beech was confirmed because the tested strain induced large lesions on the beech saplings.

## Introduction

European beech (*Fagus sylvatica* L.) is one of the major tree species in Central Europe and has an important impact on the ecology and hydrogeology of forests (Danti et al., 2002). For example it is the most important broadleaved tree species in natural woodland vegetation and managed forests in Germany (BMEL, 2018). Beech forests are characteristic elements of the potential natural zonal vegetation of Germany (Tüxen and Preising, 1956; Ellenberg, 1996; Bohn et al., 2003), which means, that these vegetation types would prevail under current environmental conditions in the absence of human intervention. The German temperate, four-season climate is currently characterized by pronounced cold winters, generally overcast with limited precipitation, and warm summers. The latter are associated with a clear west–east cline in temperature. Therefore, summers vary regionally from hot and dry to cool and rainy (Bartsch et al., 2020). In recent decades, as a result of global warming, in Germany and worldwide, there has been a general trend toward higher temperatures [Deutscher Wetterdienst (DWD), Friedrich and Kaspar, 2019; Kaspar and Friedrich, 2020]. In Germany, the absolute annual mean temperature according to the Berkeley Earth averaging% method is 8.3°C. The linear temperature trend over the period 1881 to 2019 was + 1.6°C (Kaspar and Friedrich, 2020). The year 2018, with a mean temperature of 10.5°C, was the warmest year recorded in Germany since the start of systematic observations in 1881 (Friedrich and Kaspar, 2019), followed by the years 2020 (10.4°C, Imbery et al., 2021) and 2019 (10.3°C, Kaspar and Friedrich, 2020). In comparison to the international reference period 1961–1990, the annual mean temperature in 2018 was +2.3°C higher (Friedrich and Kaspar, 2019). In addition, these three years were characterized by pronounced spring drought (Imbery et al., 2021) which, together with the extraordinary weather conditions throughout most months, resulted in root zone soil moisture and soil water storage deficits (Eichhorn et al., 2020; Tijdeman and Menzel, 2021). According to DWD, in 2018 the total annual precipitation was 590 l/m^2^ in Germany, regionally ranging from 250 – 1800 l/m^2^. As a result, the total precipitation across Germany in 2018 reached only 75% of the long term average (789 l/m^2^) relative to the reference period 1961–1990. In the year of our study, 2020, the total precipitation across Germany was 710 l/m^2^ (a deficit of -10%) and in Lower Saxony it was only 689 l/m^2^ (a deficit of -13%). Generally, periods of soil moisture drought stress can last longer than the meteorological drought that causes them and can have negative effects on plants or tree growth and health (Eichhorn et al., 2020; Tijdeman and Menzel, 2021).

Even though European beech has a wide climatic and geological amplitude, as well as high shade tolerance and growth capacity (Ellenberg, 1996; Bartsch et al., 2020), stands of it face loss of vitality due to ongoing climate change. This is because the climatic amplitude of this Atlantic to Subcontinental tree species, i.e., 4 – 12°C mean annual temperature and 450 – 2000 l/m^2^ rainfall Kölling (2007), reaches its limits regionally. The extraordinary weather conditions in the years 2018 to 2020 (Eichhorn et al., 2020; Imbery et al., 2021), including prolonged periods of high temperature, droughts and high levels of solar radiation, increased the vulnerability of European beech to fungal infection and beetle attacks, and triggered sun-scorch and the outbreak of complex diseases mainly caused by latent fungal pathogens (Langer et al., 2020). Loss of vitality and mortality of European beech have been observed and studied in several regions of Germany (Langer et al., 2020). In these studies, a number of fungi associated with woody tissues of European beech have been identified or isolated. Some of these were already known to be typical endophytes of beech woody tissues, such as *Asterosporium asterospermum* (Pers.) S. Hughes, *Apiognomonia errabunda* (Roberge ex Desm.) Höhn., *Biscogniauxia nummularia (Bull.)* Kuntze, *Hypoxylon fragiforme* (Pers.) J. Kickx f., and *Neonectria coccinea* (Pers.) Rossman and Samuels (Hendry et al., 2002; Parfitt et al., 2010; Senanayake et al., 2018; Langer et al., 2020). Some, however (e.g., *Botryosphaeria corticola* A.J.L. Phillips, A. Alves, and J. Luque), were found for the first time to be associated with the woody tissues of beech, or represented the first discovery on beech in Germany [*Sphaeropsis sapinea* (Fr.) Dyko and B. Sutton] (Langer et al., 2020).

Endophytes of *F. sylvatica* have previously been studied by several authors (e.g., Sieber and Hugentobler, 1987; Danti et al., 2002; Hendry et al., 2002; Baum et al., 2003; Sieber, 2007; Langer et al., 2021). Langer et al. (2020) examined an outbreak of Vitality Loss of European Beech (VLB) and found that endophytes, especially latent pathogens in the families Botryosphaeriaceae, Nectriaceae, Diaporthaceae and Xylariales, had a negative impact on tree health under the extreme weather conditions already mentioned.

In this study, 12 ascomycetous fungi associated with woody tissues of European beech were examined to shed light on the beech endophyte community and examine the impact on tree health. The aims of this study were (1) to confirm the pathogenicity and virulence in planta of latent fungal pathogens originally associated with bark necrosis or VLB, (2) to test virulence of the fungal species under field conditions in vital trees not exposed to drought stress, and (3) to determine whether there are interactions between beech stem endophytes and the inoculated pathogens. Therefore, the virulence of these endophytes in relation to the production of bark necroses and as potential pathogens of European beech was tested in inoculation tests involving potted beech saplings growing under field conditions.

## Materials and Methods

Twelve ascomycetous fungal strains originating from two different host species (*Acer pseudoplatanus* L. and *Fagus sylvatica* L.) sampled in Germany (Table 1) were tested on 137 6 – 17 year-old (mean: 9.6 years old, estimated by year ring analysis of 20 saplings) potted *Fagus sylvatica* plants in a field study.

**TABLE 1:** Pathogenicity tests: test strains, necrosis length, results of re-isolation.

**Species**	**Strain**	**Accession No.**	**Year of isolation**	**Original host tree**	**Host tissue**	**Necrosis length (mm, arithmetic mean of nine studied trees)**	**Reaction of host tissue**	**Re-isolation from inoculated tissue after 4 months**		
								**Re-isolation of test species**	**isolated filamentous morphotypes (MTs)**	**additional isolated filamentous fungi**
*Biscogniauxia mediterranea*	NW-FVA 5283	MT561410	2019	*F. sylvatica*	wood of twig with dieback symptoms	11.3	Necrotic lesions visible, mostly no callus formation	yes	3	e.g., *N. coccinea*
*Biscogniauxia nummularia*	NW-FVA 5282	MT561409	2019	*F. sylvatica*	wood of twig with dieback symptoms	13.9	Necrotic lesions visible, no callus formation	yes	2	*N. coccinea*
*Botryosphaeria corticola*	NW-FVA 4897	MN698983	2019	*F. sylvatica*	fruiting body on bark	69.8*	Large Necrotic lesions visible, no callus formation, orange-brownish discolorations in the whole stem	yes	1	none
*Botryosphaeria dothidea*	NW-FVA 5287	MZ045841	2019	*Fagus sylvatica*	wood trunk	11.6	Necrotic lesions visible, T-shaped discoloration, inoculation locus of some trees overgrown by callus formation,	yes	3	e.g., *N. coccinea*
*Botryosphaeria stevensii*	NW-FVA 4915	MN698981	2019	*F. sylvatica*	wood trunk	12.8	Necrotic lesions visible, no or minor callus formation	yes	4	e.g., *N. coccinea, Sordaria* sp.
*Eutypella quaternata*	NW-FVA 5333	MZ045842	2019	*F. sylvatica*	ascocarp on bark	19.3	Necrotic lesions visible, no callus formation	yes	3	e.g., *N. coccinea*
*Hypoxylon fragiforme*	NW-FVA 5174	Species identical with MZ045852	2019	*Fagus sylvatica*	wood trunk	10.4	Necrotic lesions visible, with callus formation, T-shaped discoloration	yes	4	e.g., *N. coccinea*
*Nectria cinnabarina*	NW-FVA 1249	MZ045843	2012	*A. pseudo-platanus*	stem necrosis	23.0**	Necrotic lesions visible, no callus formation	yes	3	e.g., *N. coccinea*
*Neonectria coccinea*	NW-FVA 5096	MN698988	2019	*F. sylvatica*	stem necrosis	17.9	Necrotic lesions visible, no callus formation	yes	2	e.g., Zygomycota.
*Neonectria ditissima*	CBS 226.31	JF735309	1925	*F. sylvatica*	bark	10.7	Necrotic lesions visible, T-shaped discoloration, with callus formation	no	3	e.g., *E. quaternata, Clitopilus sp.* (MZ045857)
*Neonectria aff. coccinea*	NW-FVA 0179	MZ057618	2009	*F. sylvatica*	stem necrosis	16.4	Necrotic lesions visible, no callus formation	yes	3	e.g., *Fusarium* sp.
*Sphaeropsis sapinea*	NW-FVA 4932	MN698984	2019	*F. sylvatica*	wood trunk	10.7	Necrotic lesions visible, T-shaped discoloration, with callus formation	yes	5	e.g., *Diaporthe* sp., *Fusarium* sp., *Clitopilus* sp. (MZ045858)
Mock control	MYP	-	-	-		0.0	No Necrotic lesions visible, inoculation locus overgrown by callus	na	4	e.g., *H. fragiforme, Pezicula* sp.
Untreated control	untreated	-	-	-		0.0	No Necrotic lesions visible, healthy tissue	na	3	e.g., *B. mediterranea*, *H. fragiforme*

*Only eight necrotic lesions were measured, because one tree disappeared during the experiment unrelated to the inoculation test.

**Only six necrotic lesion were measured, because only seven trees were inoculated, in total. na, not applicable.

The saplings were excavated from a natural regeneration stand in March 2020 (origin: beech dominated mixed forest, Lower Saxony, Germany; 51°35′39.7″N 9°49′46.2″E). The health of the saplings appeared to be very good at the beginning of the experiment and no symptoms of Vitality loss, bark necrosis or other disease symptoms were visible. The stem height of the saplings was 169 –327 cm (mean = 260.5 cm, *n* = 137) and the stem diameter 70 cm above ground was 0.96 – 2.05 cm (mean = 1.5 cm, *n* = 137). The saplings were planted in 65 l black plant pots containing potting compost (PROFI-LINIE Kleeschulte Topfsubstrat mineralisch: pH 6, salinity 1.5 g/l, N total: 320 mg/l, P_2_O_5_: 120 mg/l, K_2_O: 350 mg/l, Mg: 120 mg/l, Kleeschulte Erden GmbH & Co. KG, Briloner Straße 14, D-59602 Rüthen, Germany). The 137 plants were randomly assigned to blocks and kept at natural air temperature (Tmean 17°C, Tmin. 4°C and Tmax. 35°C). They were watered regularly via a Drip irrigation system (Friesecke Bewässerungstechnik GmbH, Leineweberstr. 7, 31303 Burgdorf, Germany) and shaded by surrounding older trees. During the experiment the watering regime was adjusted to supply the saplings with the optimal amount of water and air temperatures were measured by a HOBO^®^ Pro v2 data logger. The endophyte community in the woody tissues of the plant material collected from the natural beech regeneration site was determined prior to the experiment. Three saplings were examined to determine whether there was pre-colonization of the internal tissues by the test species and to profile the endophyte community. After surface disinfection with a 70% ethanol spray, stem bark and small wood chips (89 inocula, max. 0.5 × 0.5 cm in size) were obtained and placed in Petri dishes with culture medium, then incubated at room temperature in daylight. They were cultivated on malt yeast peptone agar (MYP), modified according to Langer (1994), containing 0.7% malt extract (Merck 1.05391.0500, Darmstadt, Germany), 0.05% yeast extract (Fluka 70161-100G, Seelze, Germany), 0.1% peptone (Merck 1.07272.0500), and 1.5% agar (Fluka 05040-1KG). The Petri dishes were checked periodically for emerging mycelia, which were subsequently sub-cultured on fresh MYP medium to obtain pure cultures. Isolated, representative fungal strains were kept on MYP slants at 4°C.

Following Bußkamp et al. (2020), isolated endophytic strains were assigned to morphotypes and identified on the basis of micro-morphological characters and/or sequencing of the ITS1-5.8S-ITS2 region of rDNA (White et al., 1990). At least one representative strain of each morphotype was used for molecular identification, involving DNA extraction from the mycelium, following Izumitsu et al. (2012), then PCR and sequencing of the target region. Sequences obtained were aligned and manually edited using MEGA5 software (Tamura et al., 2011), then used in BLAST searches of the GenBank databases (Altschul et al., 1997^1^) to identify similar ITS sequences. A similarity threshold of 98% was set for species-level identification. Generally, morphological characteristics were used to confirm the results of molecular identification.

With the exception of *Neonectria ditissima* (CBS 226.31) all fungal strains used in our pathogenicity tests were isolated from material associated with cases of disease from Germany that have been studied by the NW-FVA. Apart from *Nectria cinnabarina* (NW-FVA 1249, isolated from sycamore in Lower Saxony) all tested strains were isolated from woody tissues or bark necrotic tissue of diseased European beech trees following the procedure described above for endophytes from fruiting bodies growing on these trees. They were identified based on morphology and ITS sequencing. The ITS sequences (containing ITS1, 5.8S, and ITS2 regions) have been deposited in GenBank (Table 1) and all of the strains are permanently stored in the NW-FVA strain collection. The test strains (Table 1) included species of *Botryosphaeriaceae* (*Botryosphaeria corticola*, anamorph: *Diplodia corticola A.*J.L. Phillips, A. Alves and J. Luque, NW-FVA 4897: Hesse); *B. dothidea* (Moug.) Ces. and De Not., anamorph: *Fusicoccum aesculi* Corda, NW-FVA 5287: Saxony-Anhalt; *B. stevensii* Shoemaker, anamorph: *Diplodia mutila* (Fr.) Mont., NW-FVA 4915, Hesse; and *Sphaeropsis sapinea*, synonym: *Diplodia sapinea* (Fr.) Fuckel (teleomorph not known, NW-FVA 4932, Hesse), *Diatrypaceae* [*Eutypella quaternata* (Pers.) Rappaz, anamorph: *Libertella faginea* Desm., NW-FVA 5333, Hesse], *Graphostromataceae* [*Biscogniauxia mediterranea* (De Not.) Kuntze, NW-FVA 5287 and *B. nummularia* (Bull.) Kuntze, NW-FVA 5282, both with *Nodulisporium*-like anamorph and collected in Saxony-Anhalt], *Hypoxylaceae* [*Hypoxylon fragiforme* (Pers.) J. Kickx f., anamorph: *Nodulisporium ochraceum* Preuss, NW-FVA 5174: Saxony-Anhalt], and *Nectriaceae* [*Neonectria ditissima* (Tul. and C. Tul.) Samuels and Rossman = *Neonectria galligena* (Bres.) Rossman and Samuels, anamorph: *Cylindrocarpon heteronema* (Berk. and Broome) Wollenw. = *Cylindrocarpon willkommii* (Lindau) Wollenw.: Saxony; *Neo. coccinea*, anamorph: *Cylindrocarpon candidum* (Link) Wollenw., NW-FVA 5096: Hesse; *Neo.* aff. *coccinea*, NW-FVA 0179, Schleswig-Holstein), and *Nectria cinnabarina*, anamorph: *Tubercularia vulgaris* Tode: Schleswig-Holstein].

To estimate the impact of the test strains on European beech health, pathogenicity tests were conducted according to Henle-Koch postulates (Evans, 1976) and necrosis length was measured. The inoculation experiments were performed at the nursery of the NW-FVA in Hann. Münden, southern Lower Saxony, Germany (51°25′38.7″N 9°38′18.6″E) from 26.05.2020 until 30.09.2020 (4 months). For each tested strain (except *N. cinnabarina*), ten saplings were inoculated with an MYP-agar plug of a one-week-old culture of the fungus. The plugs were placed in wounds made with a sterile cork borer (5 mm diameter) in the stem at a height of 70 cm. The removed bark was replaced on top of the plug, then the stem was wrapped with Parafilm. A set of 10 untreated controls, trees which were not inoculated at all, and mock controls, prepared by inoculating 10 trees with a sterile pure culture medium plug of MYP, were created. After 4 weeks (29.06.2020) one sapling per treatment group was sampled to check infection success and necrosis formation. The bark around the area of inoculation was peeled away to allow us to see and measure the extent of any necroses. Lesion lengths were measured with a ruler in the vertical direction to an accuracy of 1 mm. Fungi were re-isolated from discolored and non-discolored tissue and the resulting isolates were identified. The pathogenicity tests ended four months after inoculation with the twelve potential latent pathogens. The impact on tree health of each strain was estimated on the basis of the size of the necrotic lesions. In addition, the reaction of the host tissue, such as healing over, formation of a callus, or T-shaped discolorations, was recorded. The latter are dark or black colored T-shaped defects/discoloration of the wood, visible in a cross-section of the stem, caused by healing over of a wound or bark necrosis. Statistical differences in length of the necrotic area between different strains and controls were investigated using ANOVA and Tukey’s HSD *post hoc* test. All data analysis was conducted in R, version 3.6.2 (R Core Team, 2019). Differences were considered statistically significant if their p-value was below the threshold of 0.01. To confirm the infection and to guarantee that Koch’s postulates were fulfilled, pieces from the interface between necrotic and healthy tissue of one sapling collected in June and 3 – 7 saplings collected in September from each treatment group were surface sterilized (as described above) and plated on MYP agar. In total, 87 tissue samples, an average of 6.9 inocula per tested tree, were incubated in June (one month post inoculation) and 306 samples, an average of 5.9 inocula per tested tree, in September (four months post inoculation) were incubated for 4 weeks in an effort to re-isolate the inoculated strain. Re-isolated fungi were identified morphologically.

## Results

### Pre-experiment – Isolation of Beech Stem Endophytes

In total, we were able to isolate 18 filamentous, endophytic fungal species from the 89 inocula retrieved from stem bark and wood of the three beech saplings sampled at the beginning of the study (Table 2). Three species were isolated from all three studied trees: *Diaporthe* sp. 2, *Hypoxylon fragiforme*, and *Coniochaeta* sp. Three species were isolated from two saplings: *Chaetomium* sp. 1, *Diaporthe* sp. 1, and *Talaromyces* sp. Twelve fungi were found in only one of the three saplings: ascomycetous species 1_5481, ascomycetous species 2_5482, ascomycetous species 3_5484 and ascomycetous species 4_5490, *Chaetomium* sp. 2, *Coprinellus micaceus*, *Diaporthe* sp. 3, *Hypoxylon rubiginosum* (Pers.) Fr., *Jackrogersella cohaerens* (Pers.) L. Wendt, Kuhnert and M. Stadler, *Lepteutypa fuckelii* Petr., *Nigrospora oryzae* (Berk. and Broome) Petch, and *Penicillium glandicola* (Oudem.) Seifert and Samson. All the species that were shown to be present as endophytes, with the exception of the white-rot fungus *Coprinellus micaceus* (Bull.) Vilgalys, Hopple and Jacq. Johnson, Agaricaceae Basidiomycota, were members of the Ascomycota. Only one of the test species for the inoculation tests, *H. fragiforme*, was isolated as an endophyte from beech stems.

**TABLE 2:** Pre-isolation of stem woody and bark tissues of three *Fagus sylvatica* saplings.

**NW-FVA No.**	**Accession No.**	**Species**	**Best match blast 23.04.2021**	**Accession No.**	**Sapling 1**	**Sapling 2**	**Sapling 3**
5481	na	ascomycetous species 1	Not amplified		1		
5482	na	ascomycetous species 2	Not amplified				1
5485	na	ascomycetous species 3	Not amplified				1
5490	MZ045844	ascomycetous species 4	*Lophiostoma corticola*	KU712227	1		
5466	MZ045845	*Chaetomium* sp.1	*Chaetomium* sp.	MN341311	1		1
5471	MZ045846	*Chaetomium* sp. 2	Fungal sp.	KF212321		1	
5467	MZ045847	*Coniochaeta sp.*	*Coniochaeta* sp.	MT644317	1	1	1
5478	MZ045848	*Coprinellus micaceus*	*Coprinellus micaceus*	MF161199			1
5458	MZ045849	*Diaporthe* sp. 1	*Diaporthe viticola*	KC145906	1		1
5473	MZ573778	*Diaporthe* sp. 2	*Diaporthe eres*	MH931269	1	1	1
5486	MZ045851	*Diaporthe* sp. 3	*Diaporthe melonis*	MW308548	1		
5465	MZ045852	*Hypoxylon fragiforme*	*Hypoxylon fragiforme*	EF155528	1	1	1
5494	MZ045853	*Hypoxylon rubiginosum*	*Hypoxylon rubiginosum*	MT214998			1
5492	MZ045854	*Jackrogersella cohaerens*	*Annulohypoxylon cohaerens*	EF026140	1		
5470	MZ045855	*Lepteutypa fuckelii*	*Lepteutypa fuckelii*	NR_154123.1		1	
5460	MZ045856	*Nigrospora oryzae*	*Nigrospora oryzae*	MG098254	1		
5461	na	*Penicillium* sp.	Not amplified			1	
5472	MZ045914	*Talaromyces* sp.	*Talaromyces verruculosus*	MH398576		1	1
In total: 18 Morphotypes				sum	10	7	10

*na, not applicable.*

### Pathogenicity Tests

Four weeks post inoculation, necrosis formation was not obvious in any of the trees inoculated with the test strains. In untreated and mock controls no necrotic lesions were visible and no fungi could be isolated from the beech tissue in the area of inoculation. Apart from *Neonectria ditissima*, all the species used for inoculation could be isolated from the inoculated saplings four months after treatment (Table 1). In addition to the inoculated species, ten other fungi associated with the samples were isolated, these can be regarded as naturally occurring endophytes (Table 1). Three different fungal endophytes, including *Bi. mediterranea* and *H. fragiforme* were isolated from the untreated controls. From the mock-controls, four different fungal species were isolated, including *H. fragiforme.* In both control types no strains of the other inoculated species were shown to be present as endophytes. No additional endophytic species were isolated from the saplings inoculated with *Bo. corticola*, while from the saplings inoculated with *Neo. coccinea* only Zygomycota strains were recorded. *Neo. coccinea* was also isolated from the saplings inoculated with *Bi. mediterranea, Bi. nummularia, Bo. dothidea, Bo. stevensii, E. quaternata, H. fragiforme*, and *Nec. cinnabarina*. *E. quaternata* and *Clitopilus* sp. (Basidiomycota) were isolated from the saplings inoculated with *Neo. ditissima.* As well as *Fusarium* sp. and *Diaporthe* sp., the same *Clitopilus* sp. was isolated from the saplings inoculated with *S. sapinea.*

The months during which the field experiment took place were significantly warmer in Germany than the long-term mean for the international reference period 1961–1990 (NW-FVA, 2021). The mean air temperature of Lower Saxony was 16.6°C (+ 2.1 compared to the mean) in June, 17.5°C (−0.1°compared to the mean) in July, 20.1°C (+ 3.6°compared to the mean) in August, and 14.5°C (+ 1°compared to the mean) in September. During the pathogenicity tests the mean air temperature was 17°C and 42 days with air temperatures over 25°C, including 8 days ≥ 30°C were recorded at Hann. Münden. The total mean precipitation in Lower Saxony was 25 l/m^2^ (−59% compared to the mean) in May, 16.6°C (−8% compared to the mean) in June, 65 l/m^2^ (−11% compared to the mean) in July, 65 l/m^2^ (−7.1% compared to the mean) in August, and 45 l/m^2^ (25% of the mean) in September.

**FIGURE 1:**
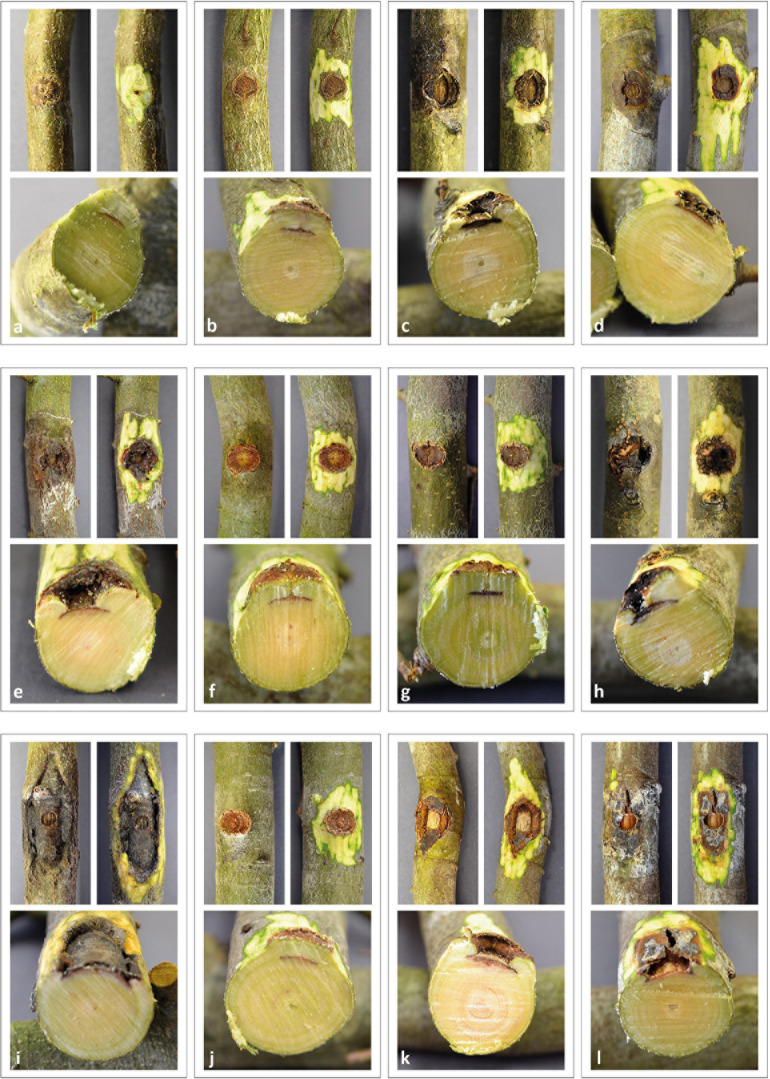
Pathogenicity tests – Necrosis induced in saplings of European beech inoculated by different fungi. **(a)** Mock control, **(b)** *Hypoxylon fragiforme*, **(c)** *Biscogniauxia mediterranea*, **(d)** *Biscogniauxia nummularia*, **(e)** *Eutypella quaternata*, **(f)** *Sphaeropsis sapinea*, **(g)** *Botryosphaeria dothidea*, **(h)** *Botryosphaeria stevensii*, **(i)** *Botryosphaeria corticola*, **(j)** *Neonectria ditissima*, **(k)** *Neonectria coccinea*, **(l)** *Nectria cinnabarina*.

**FIGURE 2:**
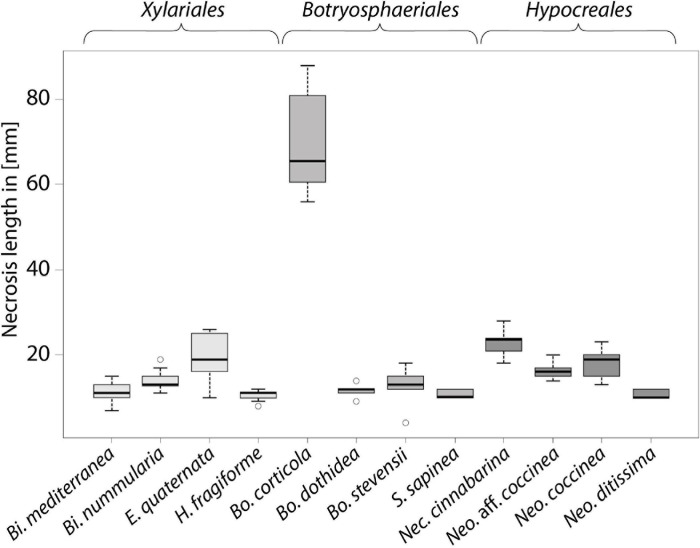
Length of necrosis induced in saplings of European beech inoculated by different fungi, boxplot of lesion length.

At the end of the experiment, all saplings were still alive but exhibiting different reactions to the various treatments (Table 1 and Figures 1, 2). In the untreated controls, no lesions were visible and the tissues were healthy. In the tissue surrounding the inoculation areas of the Mock control no lesions were visible either and the inoculation locus was overgrown by callus. All inoculated strains caused necrotic lesions with or without callus formation and with or without T-shaped discolorations. Trees inoculated with *B. corticola* exhibited the largest necrotic areas, and these were significantly larger than all other lesions (68 mm arithmetic mean necrosis length, Figure 2) and no callus formation had occurred within four months. In addition, in these saplings an orange-brownish discoloration was visible in the whole stem. The second longest lesions were present on saplings inoculated with *Nec. cinnabarina* (23 mm), followed by saplings inoculated with *E. quaternata* (19.3 mm), *Neo. coccinea* (17.9 mm), *Neo*. aff. *coccinea* (16.4 mm), *Bi. nummularia* (13.9 mm), *Bo. stevensii* (12.8 mm), *Bo. dothidea* (11.6 mm), *Bi. mediterranea* (11.3 mm), *Neo. ditissima* (10.7 mm), *S. sapinea* (10.7 mm), and *H. fragiforme* (10.4 mm). Occlusion of the wounds caused by the cork borer was observed in some or all treated saplings inoculated with *Bi. mediterranea*, *Bo. dothidea*, *Bo. stevensii*, *H. fragiforme, Neo. ditissima, and S. sapinea* within four months. As well as for *Bo. corticola*, no healing over within four months was recorded in the saplings inoculated with *Nec. cinnabarina, Neo. coccinea*, *Neo*. aff. *coccinea, Bi. nummularia*, and *E. quaternata.* T-shaped discolorations were visible in saplings inoculated with *Bo. dothidea, H. fragiforme, Neo. ditissima, and S. sapinea.*

## Discussion

With the exception of the mock-controls, all inoculations with fungal mycelium via wounding led to the presence of necrotic lesions. In some cases wounds with fungal inoculation were able to heal completely or nearly so within four months; the species for which this happened included *Bi. mediterranea, H. fragiforme, Neo. ditissima*, and *S. sapinea.* This suggests a lower virulence compared to the other test strains. These fungal strains could, at least, be quiescent colonizers functioning as endophytes after the wounds have healed. Our results showing that all of the tested fungal species induced necrosis in the saplings are not surprising because they are known to be latent pathogenic wood-decaying fungi of *F. sylvatica* or common pathogens of other tree species. Nevertheless, a single stem infection via wounding and inoculation of a beech sapling with the test species was not sufficient to kill the whole the tree within the four months of the experiment. With the exception of *Neo. ditissima*, all test strains could be re-isolated, indicating successful infection by these strains and establishment in the woody tissue. In the following, the fungi examined are discussed mainly in the order of induced necrosis length.

### Botryosphaeria corticola

The most prominent and longest necrotic lesions were caused by *Bo. corticola*, which differs from the other tested Botryosphaeriaceae in its virulence with regard to *F. sylvatica*. *Bo. corticola* is a latent plant pathogen which has been isolated at high frequency from physiologically impaired trees (Panzavolta et al., 2017). It is associated with oak decline in the Mediterranean area (Linaldeddu et al., 2014; Panzavolta et al., 2017) and causes cankers on stems, branches and twigs of oak trees (“Bot canker of oak,” Dreaden et al., 2011) and dieback of oak (Smahi, 2017). As well as its distribution in the Iberian Peninsula, Italy, Algeria, and North America (Alves et al., 2004; Dreaden et al., 2011; Smahi, 2017), *Bo. corticola* was recently found on *F. sylvatica* in Germany (Langer et al., 2020). The *Bo. corticola* test strain used in our study was the first record of this species in Germany; it was isolated from diseased mature European beech, causing bleeding spots and bark necroses (Langer et al., 2020). Preliminary investigations on pathogenicity and virulence of Botryosphaeriaceae in relation to cut branches of European beech saplings in a greenhouse experiment were performed by Langer and Bußkamp (2021). They showed that necrosis length due to inoculated Botryosphaeriaceae was positively correlated with the lesion width. Within three weeks of incubation at a mean temperature of 25°C, the length of necrotic lesions caused by *Bo. corticola* (mean: 58.3 mm) was smaller than those caused by *Bo. stevensii* (mean: 73.1 mm), while branches inoculated with *S. sapinea* showed no visible lesions. The results of our study suggest that with an adequate water supply and the mean air temperature of 17°C maintained during the experiment, *B. corticola* is significantly more virulent as a wound pathogen than *Bo. stevensii.* The inoculation experiments in planta showed, that *Bo. corticola* can be a highly virulent wound pathogen of European beech. If it should turn out that *Bo. corticola* is a frequent endophyte of European beech, there is an increased risk in the context of climate change or global warming, since it is a weak pathogen with optimal growth *in vitro* at 25°C (Smahi, 2017) and it is able to cause large necrotic lesions during in planta experiments and causes bark necrosis and bleeding spots under natural conditions. In the context of Vitality loss of European beech (VLB, Langer et al., 2020), *Bo. corticola* may play an important role as one of the first major fungal causal agents in the disease progression.

### Nectria cinnabarina

*Nectria cinnabarina*, also known as coral spot (Line, 1922), is a weak pathogen and later a saprophyte of Beech. Its ability to cause cankers and dieback on broadleaved trees (Line, 1922; Hirooka et al., 2011) was confirmed by our results: on saplings inoculated with *Nec. cinnabarina*, the second longest necrotic lesions and no healing over were observed within four months. Normally, *Nec. cinnabarina* invades hosts via wounds by spreading through the wood cells from dead tissue into the living xylem. Xylem is blocked by the fungal hyphae, leading to wilting and death of all parts above the infection site (Line, 1922). However, whether the necroses were really caused by *Nec. cinnabarina* alone is questionable, since *Neo. coccinea* was also isolated in the area of the inoculation.

### Eutypella quaternata

*Eutypella quaternata* is a common bark fungus on European beech in Germany, fruiting on dead branches (Vasilyeva and Scheuer, 1996; Vasilyeva, 2011) and dead bark on the living stem. This fungus is considered to be a weak parasite and saprobiont and often occurs together with *Neo. coccinea* (Langer and Bußkamp, 2021). Successful infection by *E. quaternata* of the studied beech saplings was confirmed by re-isolation and, astonishingly, it produced the third longest necrotic lesions in the entire experiment. Whether the lesions were really caused by *E. quaternata* alone is questionable, since *Neo. coccinea* was also isolated in the area of the inoculation. It is conceivable that the inoculation and infection with *E. quaternata* led to the transition of *Neo. coccinea* from its latent, dormant phase to the active, parasitic phase, with the two species together causing necrosis. The two species could have a positive synergistic influence on each other with regard to their ability to cause necrosis, since the lesions observed were significantly longer than on the saplings that only had an infection with *Neo. coccinea. E. quaternata* was also isolated from saplings inoculated with *Neo. ditissima.* It can be assumed that *E. quaternata* is a xylariaceous endophyte of beech, waiting to decompose the host cell wall compounds when its hosts are senescent or exhibit poor vigor (Petrini and Petrini, 1985; Whalley, 1996).

### *Neonectria coccinea* and *Neo.* aff. *coccinea*

The xylem endophyte *Neo. coccinea* is one of the most common fungi that remains latent in healthy woody tissues of *Fagus* in Europe (Chapela and Boddy, 1988; Chapela, 1989; Castlebury et al., 2006). We, therefore, expected *Neo. coccinea* to be found as an endophyte in the pre-experiment; this was not the case. This fungus was, however, found to be associated with the necrotic tissues in almost all other pathogenicity tests with other inoculated fungi, although it was not isolated from the stem tissues of the saplings in the pathogenicity tests with *Bo. corticola* or *Neo. ditissima*, or from the mock-controls and untreated controls. The growth of *Neo. coccinea* thalli is probably inhibited by low oxygen and/or nutrient availability (Sieber, 2007; Rodríguez et al., 2011). *Neo. coccinea* is one of the key fungal causal agents of BBD (Langer, 2019) and VLB (Langer, 2019; Langer et al., 2020; Langer and Bußkamp, 2021), whereas *Neo. ditissima* takes over this function in the progression of BBD in North America (Castlebury et al., 2006). *Neo. coccinea* can also cause necrotic lesions in the bark of beech branches without previous colonization by the beech-bark scale *Cryptococcus fagisuga* Lindinger (Niesar et al., 2007; Langer et al., 2020). According to Castlebury et al. (2006), there are additional, undescribed *Neonectria* species related to *Neo. coccinea* and *Neo. punicea* (J.C. Schmidt) Castl. and Rossman occurring on beech in Europe. The tested strain, NW-FVA 0179, was intermediate between the two latter species with respect to its ITS region. For further studies, we consider that it will be crucial to determine the *Neo.* aff. *coccinea* involved more precisely using the methods of Karadžić et al. (2020). Both test strains NW-FVA 5096 and NW-FVA 0179 caused necrotic lesions of more less equal size, no callus formation was observed within four months and no other typical necrosis pathogens could be found during re-isolation. We, therefore, assume that strains of the *Neo. coccinea* group have a high capacity to cause necrosis and may lead to tree mortality. Under conditions associated with global warming, increased damage caused by *Neo. coccinea* is to be expected, since strains of this species exhibit robust, rapid mycelial growth at 30°C *in vitro* (Hirooka et al., 2013).

Under the environmental conditions encountered in our experiment, inoculation via wounding and application of mycelium do not lead to symptomless infection of the samplings. However, we also isolated *Neo. coccinea* from the necrotic areas in our pathogenicity tests involving other fungi (*Bi. mediterranea, Bi. nummularia*, *Bo. dothidea*, *Bo. stevensii*, *E. quaternata, H. fragiforme*, and *Nec. cinnabarina*) because the species is able to overgrow necrotic tissues induced by other fungi (Niesar et al., 2007) after activation of a dormant propagule The reason why we were unable to isolate *Neo. coccinea* as an endophyte in the pre-experiment remains unclear. We also still need to determine whether the additional species also isolated have an influence on the virulence of the inoculated species and which fungus, the inoculated strain or the endophyte such as *Neo. coccinea, H. fragiforme* or *E. quaternata* induced the necrosis. It should be noted here that many distinct genotypes of *H. fragiforme and Neo. coccinea* have been found to be present within small areas in the woody tissues of their hosts (Hendry et al., 2002). According the latter authors, the development of fungal species after the sapwood of their hosts has been injured or subjected to water stress is mainly influenced by the low oxygen tension persisting after damage. The main factor that stimulates the active growth of latent fungal units in their host tissues appears to be aeration (Hendry et al., 2002). Species that are known to be early colonizers, such as *Bi. nummularia, H. fragiforme*, and *Neo. coccinea*, can tolerate low oxygen tension and are selectively favored by these environmental conditions. The balance between endophytic strains and inoculated strains is affected by temperature. While the occurrence of *Bi. nummularia* and *H. fragiforme* was evenly distributed at temperatures of 25°C, *Bi. nummularia* occurred more frequently at higher temperatures around 30°C.

### *Biscogniauxia nummularia* and *Bi. mediterranea*

*Biscogniauxia nummularia* is one of the most common endophytes and latent pathogens of woody tissues of European beech (Chapela and Boddy, 1988; Chapela, 1989). The results of our pathogenicity tests revealed the ability of *Bi. nummularia* to cause necrosis on European beech in planta, which fits the results of Nugent et al. (2005) and observations in nature (Langer and Bußkamp, 2021). The inoculated saplings supplied with sufficient water exhibited necrosis, even though water deficit can be a main factor to predispose the plant to disease (Boyer, 1995). Derived from healthy functional beech xylem tissue, *Bi. nummularia* can take advantage of altered host physiology and cause strip-cankers, bark blisters, bark ruptures, and wood-decay on European beech in nature (Hendry et al., 1998; Nugent et al., 2005). The growth and virulence of xylariaceous wood-decay fungi is favored by warm temperatures (Hendry et al., 2002) and prolonged summer drought (Luchi et al., 2015). *In vitro*, *Bi. nummularia* is capable of rapid growth at 30°C (Hendry, 1993). In nature, regional and local distributions of beech trees bearing lesions caused by *Bi. nummularia* correlate with environmental conditions with low rainfall or high temperatures (Hendry et al., 2002). One explanation for the fact that the necrotic lesions caused by *Bi. nummularia* are significantly larger than those due to *Bi. mediterranea* could be that *F. sylvatica* is less sensitive to *Bi. mediterranea*, which is primarily an oak endophyte (Linaldeddu et al., 2011; Ghasemi et al., 2019) and latent serious oak pathogen (Jurc and Ogris, 2006; Mirabolfathy, 2013). In the case of inoculation with *Bi. mediterranea*, the sapling started wound-healing, whilst the saplings inoculated with *Bi. nummularia* did not form callus tissue to overgrow the wood and necroses. Our results on the virulence of *Bi. nummularia* against beech reinforce the assessment of Luchi et al. (2015) that this endophyte and latent fungal pathogen is a potential bioindicator of European beech health. The relative success of this species in colonizing large volumes of wood within beech stems *in vivo* is strongly influenced by abiotic conditions, mainly high temperatures (Hendry et al., 1998). When climatic or host conditions change, the widely distributed endophyte of European beech, *Bi. nummularia*, can cause dramatic loss of trees and stands, wood-decay, and wood devaluation, thus also weakening trees and adversely affecting occupational safety in the affected beech stands.

*Bi. mediterranea* is considered a secondary pathogen causing the disease only in stressed hosts (Henriques et al., 2015), especially those suffering from drought stress (Mirabolfathy, 2013). In Germany, *Bi. mediterranea* has been found in association with shoot dieback, necrosis and wood decay of beech in the course of VLB (Langer et al., 2020; Langer and Bußkamp, 2021). The lifestyle switch to pathogenic behavior is often associated with prolonged periods of stress of the host tree (Henriques et al., 2014). *Bi. mediterranea* is able to grow rapidly in the xylem and bark host tissues and can cause necrosis, canker and wood decay leading to accelerated tree decline and death (Henriques et al., 2015). Our research confirms this information to the extent that *Bi. mediterranea* has the capacity to cause necrosis on *F. sylvatica-*saplings and is able to colonize the host tissue. This is in accordance with the results of Mirabolfathy et al. (2011), who found *Bi. mediterranea* to be virulent and causing disease symptoms on seedlings of *Quercus castaneifolia* C.A.Mey. in a similarly designed pathogenicity test. The virulence of *Bi. mediterranea* against beech under our test conditions was similar to *H. fragiforme*, *Neo. ditissima*, and *S. sapinea* but distinctly less than *Bi. nummularia* and the other test strains. Although, the two major factors – precipitation (positive correlation) and the temperature (negative correlation) – have a significant influence on the dispersal of *Bi. mediterranea* spores, host tissue colonization and disease outbreaks, necrosis formation seems to be promoted by water stress in the host tree (Capretti and Battisti, 2007). Results presented by Vannini et al. (2008) suggest that rapid endophytic growth of *Bi. mediterranea* is favored by a decrease in host water potential, a factor which was not recorded in our experiment. Drought stress of host trees intensifies the effects of charcoal disease (Ghanbary et al., 2020), but our test saplings did not suffer from water stress.

### *Botryosphaeria stevensii*, *Bo. dothidea*, and *Sphaeropsis sapinea*

The latent pathogen *Bo. stevensii* causes cankers, branch dieback, wilting, yellowing and death of leaves and stem necrosis, and can lead to tree mortality (Vajna, 1986; Alves et al., 2004; Slippers and Wingfield, 2007). The shift to its parasitic stage is mainly initiated by water deficiency in the host tissue (HMUELV Hessen, 2011) and its spread seems to be limited by low temperatures (CABI, 2019). Apart from *Bo. corticola*, *Bo. stevensii*, induced the longest necrotic lesions of all other tested Botryosphaeriaceae strains during our inoculation experiment. This partly fits the results of Langer and Bußkamp (2021), who found that *Bo. stevensii* caused larger necrotic lesions at an average temperature of 25°C than *B. corticola*. At mean temperatures less than optimal for *B. stevensii* [optimal temperature according Kuch et al. (2014): 25°C] the mean lesion length in planta due to this pathogen is less. These results lead us suggest that the virulence of *Bo. stevensii* decreases with falling temperatures and the risk of negative impact on tree health due to this species is increasing with climate warming. Previous sporadic occurrences of *Bo. stevensii* in northern Europe (CABI, 2019) and an increasing incidence in recent years in the Mediterranean region (CABI, 2019) and in Germany (HMUELV Hessen, 2011; LFE, 2016; Langer and Bußkamp, 2021) support this hypothesis. *Bo. stevensii* may play an important role as one of first fungal causal agents in the disease progression of VLB in the context of climate change.

*Bo. dothidea* is an endophyte and latent pathogen of global importance with respect to tree health (Crist and Schoeneweiss, 1975; Smith and Stanosz, 2001; Butin, 2011; Piškur et al., 2011; Abdollahzadeh et al., 2014; Marsberg et al., 2017; Langer and Bußkamp, 2021). It seems to be native to Europe (Kehr, 2004; Piškur et al., 2011). The endophytic phase or prolonged latent infection in hosts allows *Bo. dothidea* to cause disease symptoms rapidly when its hosts become stressed (Marsberg et al., 2017; Langer and Bußkamp, 2021). Decreasing water potential and drought stress can be major factors predisposing host trees to infection by *Bo. dothidea* (Crist and Schoeneweiss, 1975; Ma et al., 2001). Our results indicate that mean air temperature is probably also an important influence on the species’ ability to cause disease symptoms because at lower temperatures it produces significantly smaller lesions (Langer and Bußkamp, 2021). Because the optimal growth temperature for *Bo. dothidea* strains is in the range 27 – 30°C (Michailides and Morgan, 1992; Yan et al., 2012), we suggest that with increasing mean air temperatures in the context of climate change, more disease outbreaks due to *Bo. dothidea* are to be expected in Germany.

*S. sapinea* is a common endophyte and pathogen of pine species, widely distributed both globally (Bihon et al., 2012) and in Germany (Bußkamp et al., 2020). The association of *S. sapinea* with woody tissues of European beech has been recently reported (Zlatković et al., 2017; Langer and Bußkamp, 2021). Heat and drought seem to have an effect on the ability of *S. sapinea* to infect broadleaved trees because occurrence of this latent pathogen has also been reported from diseased woody tissues of Cork oak in Algeria (Smahi, 2017) and *Alnus glutinosa* in Germany (Bußkamp et al., 2021). We demonstrated the pathogenicity of *S. sapinea* to European beech, confirming the results reported by Zlatković et al. (2017) and Smahi (2017). The negative influence of lower temperatures on the virulence of *S. sapinea* has been suggested by Langer and Bußkamp (2021), indicating that this species was not able to induce necrosis on woody beech tissues at a mean air temperature of 11°C. The role of *S. sapinea* in the progression of VLB and other complex diseases of *F. sylvatica* in the light of climate change is far from fully understood.

### Neonectria ditissima

In Germany, *Neo. ditissima* is often associated with rough bark beech, phloem necrosis or beech canker (Grüner and Metzler, 2006) and has primarily been isolated from *Fagus, Malus* and *Salix* (Castlebury et al., 2006; Ghasemkhani et al., 2016). The results of the pathogenicity tests with *Neo. ditissima* and the pre- and post-experiment isolation of fungi associated with the stems of the beech saplings cannot be interpreted unambiguously and raise various questions. The test strain used was originally sampled in 1931 from *F. sylvatica* and still exhibited good growth *in vitro*. In contrast to the other strains used, age-related low virulence cannot be ruled out. The necrotic lesions observed on saplings inoculated with *Neo. ditissima* were significantly smaller than on saplings infected with the other Nectriaceae and healed over within four months of inoculation. Based on the results of re-isolation after inoculation, successful infection of the beech saplings is not guaranteed. When infection was successful and necrotic lesions were induced by *Neo. ditissima*, the species/strain appeared to be less virulent than the other tested Nectriaceae. Re-isolation may have failed because the additional isolated fungi, *E. quaternata* and *Clitopilus* sp. could have overgrown the lesions and *Neo. ditissima.* If the infection with *Neo. ditissima* failed, the endophyte *Eutypella quaternata* may have overcome its dormant phase and induced the lesions.

Natural infection with *Neo. ditissima* takes place by means of ascospores and conidia that settle in host wounds (Gómez-Cortecero et al., 2016). The limiting factor for infection in nature is rainfall, because wet conditions are essential for spore production, spread, germination and infection by *Neo. ditissima* (Xu and Ridout, 1998; Xu and Robinson, 2010). Xu and Robinson (2010) reported several factors affecting the incidence of canker and the incubation period, the period between infection and appearance of symptoms. Plant resistance and pathogen virulence are influenced by temperature, wetness and the time of year when inoculation takes place. The incidence of canker caused by *Neo. ditissima* decreases as the age of the wound increases. From this perspective, the initial conditions for infection and formation of necroses in our study were ideal, because the interval between wounding in our pathogenicity test and inoculation with the test strain was very short, since the mycelial plug was inserted immediately after wounding. In relation to environmental conditions and wetness during the pathogenicity test, there were rather unfavorable conditions for an infection because it was significantly warmer and drier in Germany compared to the long-term mean (NW-FVA, 2021).

### Hypoxylon fragiforme

The wood-decaying species *H. fragiforme* is a common endophytic fungus in angiosperm woody tissues (Parfitt et al., 2010) but also occurs as an endophyte in gymnosperms, especially in Scots pine (Bußkamp et al., 2020). The transient endophytic lifestyle of *H. fragiforme* originating from xylem tissue, allows this fungus to progress on to saprotrophic wood-decay activity (Parfitt et al., 2010). Next to *Bi. nummularia* and *Neo. coccinea*, *H. fragiforme* is one of the three most common latent fungi in woody tissues of beech (Chapela and Boddy, 1988; Chapela, 1989), occurring in twigs, branches, and stems of European beech (Hendry et al., 2002). Usually, *H. fragiforme* can be seen fruiting with its anamorph or teleomorph (Red cushion *Hypoxylon*) in clusters on the bark of dead beech or dead areas on living trees. The results of our pathogenicity tests confirm those reported by Hendry et al. (2002) that *H. fragiforme* is able to form necrotic lesions on beech. The lesions induced by *H. fragiforme* were significantly smaller during our experiment under elevated temperatures than caused by *Bi. nummularia.* This fits the results of Hendry et al. (2002) who found that the development of *H. fragiforme* decreased at temperatures between 25 and 30°C. *H. fragiforme* appeared to be less virulent against beech than the other *Xylariales* species and co-occurred with *Neo. coccinea*.

## Conclusion and Outlook

Latent plant pathogenic fungi of beech that exhibit endophytic behavior, such as *Neo. coccinea, Bi. nummularia, Bo. corticola, Bo. dothidea, Bo. stevensii*, and *E. quaternata*, are sensitive to changes in host physiology driven by stress conditions, as predicted by Desprez-Loustau et al. (2006). However, the relative success of these species in planta is not only strongly influenced by abiotic conditions such as water availability to the host and environmental conditions during infection (e.g., air temperature and humidity), but also biotic factors. These probably include the vigor and resistance of the host, host shift of the pathogen, the vitality, age and possible loss of pathogenicity of the tested strains during storage in artificial conditions, but also the co-occurrence of other quiescent fungal species inhabiting the host trees.

The elevated temperatures during our pathogenicity tests, which are a consequence of the current climate change in Germany, favored establishment and growth of the early-colonizing species, e.g., *Neo. coccinea, Bi. nummularia* and plant pathogens, such as *Bo. corticola, Nec. cinnabarina*, or *E. quaternata*. Latency units, mainly of *Neo. coccinea*, appeared to be activated by the host stressors wounding and/or inoculation with fungal species. The observations made in our pathogenicity tests do not allow definitive statements regarding the interactions between the co-occurring fungi and the impact on necrosis formation. Because it is not possible to obtain test saplings without endophytes, it is important in the future to carry out co-occurrence studies or antagonist tests *in vitro* with latent pathogens. In addition, any experiments should be repeated under different temperature conditions and different water availabilities for the host trees.

## Data Availability Statement

The datasets presented in this study can be found in online repositories. The names of the repository/repositories and accession number(s) can be found below: https://www.ncbi.nlm.nih.gov/genbank/, MT561410; https://www.ncbi.nlm.nih.gov/genbank/, MT561409; https://www.ncbi.nlm.nih.gov/genbank/, MT561409; https://www.ncbi.nlm.nih.gov/genbank/, MN698983; https://www.ncbi.nlm.nih.gov/genbank/, MZ045841; https://www.ncbi.nlm.nih.gov/genbank/, MN698981; https://www.ncbi.nlm.nih.gov/genbank/, MZ045842; https://www.ncbi.nlm.nih.gov/genbank/, MZ045843; https://www.ncbi.nlm.nih.gov/genbank/, MN698988; https://www.ncbi.nlm.nih.gov/genbank/, JF735309; https://www.ncbi.nlm.nih.gov/genbank/, MN698984; https://www.ncbi.nlm.nih.gov/genbank/, MZ045858; https://www.ncbi.nlm.nih.gov/genbank/, MZ045857; https://www.ncbi.nlm.nih.gov/genbank/, MZ045844; https://www.ncbi.nlm.nih.gov/genbank/, MZ045845; https://www.ncbi.nlm.nih.gov/genbank/, MZ045846; https://www.ncbi.nlm.nih.gov/genbank/, MZ045847; https://www.ncbi.nlm.nih.gov/genbank/, MZ045848; https://www.ncbi.nlm.nih.gov/genbank/, MZ045849; https://www.ncbi.nlm.nih.gov/genbank/, MZ045850; https://www.ncbi.nlm.nih.gov/genbank/, MZ045851; https://www.ncbi.nlm.nih.gov/genbank/, MZ045852; https://www.ncbi.nlm.nih.gov/genbank/, MZ045853; https://www.ncbi.nlm.nih.gov/genbank/, MZ045854; https://www.ncbi.nlm.nih.gov/genbank/, MZ045855; https://www.ncbi.nlm.nih.gov/genbank/, MZ045856; https://www.ncbi.nlm.nih.gov/genbank/, MZ045914; https://www.ncbi. nlm.nih.gov/genbank/, MZ573778.

## Author Contributions

JB organized the implementation and evaluation of the experiment and performed statistical analysis of the results. GL and JB discussed the results and prepared the manuscript. GL wrote the first draft of the manuscript. Both authors designed the concept of the research attempt and undertook identification of the fungi.

## Conflict of Interest

The authors declare that the research was conducted in the absence of any commercial or financial relationships that could be construed as a potential conflict of interest.

## Publisher’s Note

All claims expressed in this article are solely those of the authors and do not necessarily represent those of their affiliated organizations, or those of the publisher, the editors and the reviewers. Any product that may be evaluated in this article, or claim that may be made by its manufacturer, is not guaranteed or endorsed by the publisher.

## Footnotes

1
http://www.ncbi.nlm.nih.gov/genbank/
